# Genetic variants of dipeptidyl peptidase IV are linked to the clinicopathologic development of prostate cancer

**DOI:** 10.1111/jcmm.17845

**Published:** 2023-08-02

**Authors:** Yu‐Ching Wen, Chia‐Yen Lin, Chi‐Hao Hsiao, Shian‐Shiang Wang, Hsiang‐Ching Huang, Yung‐Wei Lin, Kuo‐Hao Ho, Lun‐Ching Chang, Shun‐Fa Yang, Ming‐Hsien Chien

**Affiliations:** ^1^ Department of Urology, School of Medicine College of Medicine and TMU Research Center of Urology and Kidney (TMU‐RCUK), Taipei Medical University Taipei Taiwan; ^2^ Department of Urology Wan Fang Hospital, Taipei Medical University Taipei Taiwan; ^3^ Division of Urology, Department of Surgery Taichung Veterans General Hospital Taichung Taiwan; ^4^ School of Medicine Chung Shan Medical University Taichung Taiwan; ^5^ School of Medicine National Yang Ming Chiao Tung University Taipei Taiwan; ^6^ Department of Applied Chemistry National Chi Nan University Nantou Taiwan; ^7^ Graduate Institute of Medical Sciences College of Medicine, Taipei Medical University Taipei Taiwan; ^8^ International Master/PhD Program in Medicine College of Medicine, Taipei Medical University Taipei Taiwan; ^9^ Department of Mathematical Sciences Florida Atlantic University Boca Raton Florida USA; ^10^ Institute of Medicine Chung Shan Medical University Taichung Taiwan; ^11^ Department of Medical Research Chung Shan Medical University Hospital Taichung Taiwan; ^12^ Pulmonary Research Center, Wan Fang Hospital Taipei Medical University Taipei Taiwan; ^13^ Traditional Herbal Medicine Research Center Taipei Medical University Hospital Taipei Taiwan; ^14^ TMU Research Center of Cancer Translational Medicine Taipei Medical University Taipei Taiwan

**Keywords:** clinicopathologic development, dipeptidyl peptidase IV, prostate cancer, single‐nucleotide polymorphism

## Abstract

CD26/dipeptidyl peptidase IV (DPP4) is a multifunctional cell‐surface glycoprotein widely found in many cell types, and a soluble form is present in body fluids. There is longstanding evidence indicating a tumour‐promoting or ‐suppressive role of DPP4 in different cancer types. However, studies focusing on the impacts of genetic variants of DPP4 on cancers are very rare. Herein, we conducted a case–control study to evaluate whether single‐nucleotide polymorphisms (SNPs) of DPP4 were associated with the risk or clinicopathologic development of prostate cancer (PCa). We genotyped four loci of DPP4 SNPs, including rs7608798 (A/G), rs3788979 (C/T), rs2268889 (T/C) and rs6741949 (G/C), using a TaqMan allelic discrimination assay in 704 PCa patients and 704 healthy controls. Our results showed that PCa patients with the DPP4 rs7608798 AG+GG genotype or rs2268889 TC+CC genotype had a higher risk of developing an advanced clinical primary tumour (cT) stage (adjusted odds ratio (AOR): 1.680, 95% confidence interval (CI): 1.062–2.659, *p* = 0.025; AOR: 1.693, 95% CI: 1.092–2.624, *p* = 0.018). Additionally, in The Cancer Genome Atlas (TCGA) database, we observed that lower DPP4 expression levels were correlated with higher Gleason scores, advanced cT and pathological stages, tumour metastasis, and shorter progression‐free survival rates in PCa patients. Furthermore, overexpression of DPP4 suppressed migration/invasion of metastatic PC3 PCa cells. Our findings suggest that DPP4 levels may affect the progression of PCa, and the DPP4 rs7608798 and rs2268889 SNPs are associated with the clinicopathologic development of PCa in a Taiwanese population.

## 1 INTRODUCTION

Prostate cancer (PCa) is the most common non‐skin malignancy and second leading cause of cancer deaths among men in the United States.^1^ The increasing trend of its incidence is seen more often in developed countries including Taiwan probably due to more‐advanced medical care facilities and prostate‐specific antigen (PSA) screening at early stages of disease development.^2^ Although PSA detection and a digital rectal examination (DRE) are common screening methods of clinicians for detecting PCa, these methods have limited accuracy. For example, PSA is a prostate gland‐specific antigen, but not a PCa‐specific antigen. Therefore, an elevated PSA level is also observed in benign pathologies such as prostatic hyperplasia and prostatitis.^3^ Until now, there are still no specific cancer‐associated or PCa patient‐specific biomarkers to distinguish between benign and malignant tumours. As an early diagnosis of PCa is necessary to prevent metastasis and promote treatment, research into new efficient predictive biomarkers is essential to detect at‐risk patients.

Genetic changes play important roles in the initiation and clinicopathologic development of PCa.^4^ Thus, there is a need to identify novel genetic markers which can be used as predictors for the most susceptible segments of the population to PCa or for genes that participate in PCa progression. Single‐nucleotide polymorphisms (SNPs) are variations in a genome's base pairs in a DNA sequence, with a variant frequency that occurs in more than 1% of the population and correlates with disease susceptibility, progression, or therapy responses.^5^, ^6^, ^7^ Actually, in genome‐wide association studies (GWASs) and PCa case–control surveys, an enormous number of different SNPs in different genes were found to be correlated with PCa risk or progression.^8^ For example, SNPs located in genes such as caspase‐3 (CASP3), caspase‐9 (CASP9), hepatocyte nuclear factor 1‐beta (HNF1B), cancer susceptibility 8 (CASC8) and cyclin‐dependent kinase inhibitor 1B (CDKN1B) were implicated in regulation of cell apoptosis, the cell cycle, metabolism and cell division and were shown to be correlated with PCa susceptibility.^9^, ^10^ Moreover, Y‐box binding protein‐1 (YB‐1) is a critical regulator of androgen receptor (AR) variants, which are involved in resistance to hormone therapy for PCa.^11^ The rs1203072 SNP located in an intron of the YB‐1 gene was reported to affect gene expression and be linked to PCa metastasis.^12^

DPP4, located on chromosome 2q24.3, encodes dipeptidyl peptidase IV (DPP‐IV), a 110‐kDa cell surface type II transmembrane glycoprotein. DPP4 is a multifunctional protein that not only has hyperglycemic action, but is also involved in cancer biology processes, such as migration, invasion, metastasis, apoptosis, and sensitivity to chemotherapy.^13^, ^14^ To the present, the role of DPP4 in PCa is still controversial. Wilson et al demonstrated an increase of DPP4 activity in PCa in comparison to benign prostatic hyperplasia or normal prostate tissue.^15^ Lu et al. indicated that DPP4 expression was higher in PCa tissues than in normal tissues and was correlated with the PSA level, tumour size, and cancer stage.^16^ In contrast, Russo et al. created a PCa murine xenograft model demonstrating that DPP4 is an androgen receptor‐stimulated tumour‐suppressor gene that is downregulated when the cancer progresses to castration‐resistant PCa (CRPC). In CRPC clinical samples, a decrease in the DPP4 protein was also found compared to untreated primary PCa.^17^ Moreover, Wesley et al also indicated that DPP4 inhibits the malignant phenotype of PCa cells by blocking basic fibroblast growth factor signalling pathway.^18^ In addition to the analysis of DPP4 expression level, decreased DPP4 enzymatic activity was observed in serum samples of PCa patients with metastatic disease compared to patients with localized disease.^19^ Although the clinical significance and functional role of DPP4 in PCa have been investigated by several studies, the impacts of DPP4 genetic variants on PCa remain poorly investigated. We hypothesized that functional SNPs of DPP4 may have an impact on the occurrence or progression of PCa. In this study, we intended to explore associations of SNPs within the DPP4 gene with the risk and clinicopathologic development of PCa in a Taiwanese population.

## 2 MATERIALS AND METHODS

### 2.1 Study participants

In this case–control study, we retrospectively enrolled 704 PCa patients who had received a robotic‐assisted laparoscopic radical prostatectomy (RP) at Taichung Veteran General Hospital (Taichung, Taiwan) during 2012–2018. In addition, 704 anonymized healthy male controls with the same ethnic background and living in a similar geographic area were randomly chosen from the Human Biobank of Chung Shan Medical University Hospital. Medical information of PCa patients at diagnosis was all obtained from their medical records, and included the pathologic Gleason grade, PSA values, clinical and pathologic TN (tumour, node) staging, D'Amico classification, and cancer cell invasion area (perineural, seminal vesicle and lymphovascular). Peripheral blood was collected from each participant after informed consent was obtained, and the study process was approved by the Institutional Review Board of Taichung Veterans General Hospital (IRB no. CE19062A).

### 2.2 Genomic DNA extraction from whole‐blood samples

To obtain genomic DNA, peripheral blood collected from all recruited subjects was preserved in EDTA‐containing anti‐coagulant tubes and further extracted using a QIAamp DNA Blood Mini Kit (Qiagen) according to the manufacturer's instructions. Extracted DNA was dissolved in Tris‐EDTA (TE) buffer (10 mM Tris and 1 mM EDTA; pH 7.8), and the DNA purity was further checked with a Nanodrop‐2000 spectrophotometer (ThermoFisher Scientific) to detect the ratio of absorbances at 260 and 280 nm. The final preparation was stored at −20°C before a real‐time polymerase chain reaction (PCR) analysis.

### 2.3 Selection and determination of DPP4 SNPs

In total, four SNPs in DPP4 including rs7608798 (A/G), rs3788979 (C/T), rs2268889 (T/C) and rs6741949 (G/C) were selected as these SNPs were previously reported to affect the risk, severity, or progression of other diseases and expression of the DPP4 gene.^20^, ^21^, ^22^, ^23^ Allelic discrimination of the DPP4 rs7608798 (assay ID: C_27055353_10), rs3788979 (assay ID: C_2789710_10), rs2268889 (assay ID: C_15875589_10) and rs6741949 (assay ID: C_11741570_10) SNPs was assessed using the TaqMan SNP Genotyping Assay with an ABI StepOnePlus™ Real‐Time PCR System (ThermoFisher Scientific). Detailed processes regarding DNA genotyping were published in our previous study.^24^

### 2.4 Bioinformatics analysis

The UCSC Xena database (https://xena.ucsc.edu/) provided clinical data and messenger (m)RNA sequencing of prostate adenocarcinoma (PRAD) samples from The Cancer Genome Atlas (TCGA). DPP4 gene expression levels were compared across various clinical features, such as Gleason scores, clinical stages, pathological tumour sizes, lymph nodes and distal metastases. The Wilcoxon signed‐rank test was employed for two‐group comparisons, while the Kruskal–Wallis test with post hoc Dunn's test was used for clinical features with more than two groups. The log‐rank test was utilized to assess the association between DPP4 and patients' progression‐free survival (PFS). High (DPP4^high^) and low (DPP4^low^) expression groups were determined based on the median cut‐off point of DPP4.

### 2.5 PCa cell lines and culture

The human androgen‐dependent PCa cell line, LNCaP, and androgen‐independent PCa cell lines, 22RV‐1, DU145 and PC3, were obtained from American Type Culture Collection (ATCC). DU145 cells were cultured in Dulbecco's modified Eagle medium (DMEM, Life Technologies); PC3 cells were cultured in minimum essential medium (MEM, Life Technologies); and 22RV‐1 and LNCaP cells were maintained RPMI‐1640 medium (Life Technologies). All culture media we used in this study were supplemented with 10% foetal bovine serum (FBS, Gibco‐BRL) and 1% penicillin–streptomycin–glutamine. All PCa cells were maintained in an incubator at 37°C with a 5% CO_2_ and 95% air atmosphere.

### 2.6 Transient transfection of a DNA plasmid

To overexpress DPP4 in PCa cells, 3 μg of the pENTER‐CD26 plasmid (Vigene Biosciences) was transfected into PC3 cells cultured in a 6‐mm^2^ Petri dish using Lipofectamine 3000 Transfection Reagent (Invitrogen) for 6 h according to the manufacturer's instructions. An empty vector transfected into PC3 cells was used as the control group. At 24 h after transfection, cells were analysed for DPP4 expression and migratory/invasive abilities.

### 2.7 Total protein extraction from PCa cells and Western blot analysis

Extraction of protein lysates was described previously.^25^ Concentrations of total proteins were determined using a Bio‐Rad protein assay kit (Bio‐Rad). Proteins (20–40 μg) were subjected to sodium dodecyl sulfate polyacrylamide gel electrophoresis (SDS‐PAGE) and then electrophoretically transferred to polyvinylidene difluoride (PVDF) membranes (Bio‐Rad). The DPP4 primary antibody and its horseradish peroxidase‐conjugated secondary antibody were sequentially incubated with the membrane. After washing, membranes were incubated with an enhanced chemiluminescence (ECL) Western blotting reagent, and chemiluminescence was detected with the MultiGel‐21 chemiluminescence imaging system (TOP BIO).

### 2.8 Transwell migration and invasion assays

Migration and invasion assays were performed according to our previous study.^26^ Briefly, 2 × 10^4^ PC3/vector or PC3/DPP4 cells were plated in an uncoated top chamber (24‐well insert; pore size, 8 μm; Corning Costar) for the migration assay, or 4 × 10^4^ cells were plated in the Matrigel‐coated (BD Biosciences) top chamber for the invasion assay. In both assays, the top chambers contained serum‐free medium, and medium with 10% serum used as a chemoattractant was added to the lower chamber. After allowing cells to migrate or invade for 48 h, they were fixed with methanol and stained with crystal violet. The number of cells that had migrated or invaded through the membrane was counted under a light microscope, with three fields viewed at 100× magnification used for the analysis.

### 2.9 Statistical analysis

For the association between DPP4 genotypic frequencies and clinicopathologic features, multivariate logistic regression models were used to estimate the odds ratios (ORs), adjusted ORs (AORs), and 95% confidence intervals (CIs). Statistical analyses of our data were performed with the SAS software program (vers. 9.1, 2005; SAS Institute). Statistical significance was set to *p* < 0.05.

## 3 RESULTS

### 3.1 Demographic characteristics of recruited PCa patients

Table 1 shows demographic and clinicopathological characteristics of 704 PCa patients who underwent RP. Our study population was predominantly older (57.8% were over 65 years of age). Most patients had early‐stage tumours (clinical T1 or T2 stage, 86.1%) with perineural invasion (73.6%), but without lymph node metastasis (N0, 91.5%), lymphovascular invasion (84.1%), or seminal vesicle invasion (78.6%). According to the D'Amico risk classification, more than half of the PCa patients (50.4%) had a high risk (>50% chance) of recurrence at 5 years post‐treatment.

**TABLE 1:** Distributions of demographic characteristics in 704 patients with prostate cancer.

Variable	Patients (*N* = 704)
Age at diagnosis (years)	
≤65	297 (42.2%)
>65	407 (57.8%)
PSA at diagnosis (ng/mL)	
≤10	334 (47.4%)
>10	370 (52.6%)
Pathologic Gleason grade group	
1+2+3	584 (83.0%)
4+5	120 (17.0%)
Clinical T stage	
1+2	606 (86.1%)
3+4	98 (13.9%)
Pathologic T stage	
2	372 (52.8%)
3+4	332 (47.2%)
Pathologic N stage	
N0	644 (91.5%)
N1	60 (8.5%)
Seminal vesicle invasion	
No	553 (78.6%)
Yes	151 (21.4%)
Perineural invasion	
No	186 (26.4%)
Yes	518 (73.6%)
Lymphovascular invasion	
No	592 (84.1%)
Yes	112 (15.9%)
D'Amico classification	
Low risk	83 (11.8%)
Intermediate risk	266 (37.8%)
High risk	355 (50.4%)

Abbreviations: N, node; PSA, prostate‐specific antigen; T, tumour.

### 3.2 Associations between DPP4 genetic polymorphisms and PCa susceptibility

We next investigated possible relationships of four selected SNPs (rs7608798 (A/G), rs3788979 (C/T), rs2268889 (T/C) and rs6741949 (G/C)) of the DPP4 gene with PCa susceptibility. We first analysed genotype frequencies of these SNPs in the entire population we recruited. As shown in Table 2, the highest distribution frequencies of the DPP4 rs7608798, rs3788979, rs2268889 and rs6741949 SNPs in PCa patients were heterozygous A/G and C/T for the rs7608798 and rs3788979 loci, respectively, and homozygous T/T and G/G for the rs2268889 and rs6741949 loci, respectively. Genotypic distributions of these four DPP4 SNPs in the control group conformed to Hardy–Weinberg equilibrium (χ^2^= 0.177, *p* = 0.673 for rs7608798 A > G; χ^2^ = 0.708, *p* = 0.400 for rs3788979 C > T; χ^2^ = 0.682, *p* = 0.409 for rs2268889 T > C, and χ^2^ = 0.093, *p* = 0.760 for rs6741949 G > C). We used AORs with 95% CIs estimated by multiple logistic regression models after adjusting for age to assess associations between DPP4 SNPs and PCa susceptibility. Herein, we observed no significant associations between DPP4 SNPs and PCa susceptibility in the recruited Taiwanese population, as calculated either by a dominant model or a codominant model (Table 2).

**TABLE 2:** Adjusted odds ratio (AOR) and 95% confidence interval (CI) of prostate cancer associated with DPP4 genotypic frequencies.

Variable	Controls (*N* = 704) (%)	Patients (*N* = 704) (%)	AOR (95% CI)	*p*‐Value
rs7608798				
AA	277 (39.3%)	288 (40.9%)	1.000 (reference)	
AG	325 (46.2%)	329 (46.7%)	1.124 (0.871–1.452)	0.368
GG	102 (14.5%)	87 (12.4%)	0.927 (0.638–1.347)	0.692
AG + GG	427 (60.7%)	416 (59.1%)	1.077 (0.845–1.371)	0.550
rs3788979				
CC	178 (25.3%)	193 (27.4%)	1.000 (reference)	
CT	363 (51.6%)	339 (48.2%)	0.905 (0.681–1.203)	0.491
TT	163 (23.2%)	172 (24.4%)	0.890 (0.635–1.247)	0.497
CT + TT	526 (74.7%)	511 (72.6%)	0.900 (0.688–1.178)	0.443
rs2268889				
TT	315 (44.7%)	344 (48.9%)	1.000 (reference)	
TC	319 (45.3%)	296 (42.0%)	0.955 (0.745–1.225)	0.718
CC	70 (10.0%)	64 (9.1%)	0.888 (0.583–1.352)	0.579
TC + CC	389 (55.3%)	360 (51.1%)	0.943 (0.744–1.195)	0.626
rs6741949				
GG	569 (80.8%)	578 (82.1%)	1.000 (reference)	
GC	127 (18.1%)	118 (16.8%)	0.943 (0.691–1.287)	0.714
CC	8 (1.1%)	8 (1.1%)	0.665 (0.218–2.032)	0.474
GC + CC	135 (19.2%)	126 (17.9%)	0.924 (0.682–1.251)	0.609

*Note*: AOR with the 95% confidence interval was estimated by multiple logistic regression models after controlling for age.

### 3.3 DPP4 rs7608798 and rs2268889 SNPs correlate with advanced clinical T stage of PCa patients

Next, we assessed connections of these four DPP4 genetic variants with PCa clinicopathologic features, including PSA levels at diagnosis, clinical T (cT) stage, pathologic T and N stages, tumour invasion statuses and D'Amico classification. Among the four DPP4 loci, we observed that PCa patients who carried at least one minor allele (AG and GG) of rs7608798 had a significantly higher risk of developing advanced cT stages (cT3 + 4) (OR: 1.680‐fold; 95% CI: 1.062–2.659; *p* = 0.025) compared to patients carrying the wild‐type (WT) homozygotes (AA) (Table 3). Moreover, PCa patients harbouring at least one polymorphic C allele of DPP4 rs2268889 also showed a significantly higher risk of having advanced cT stages (OR: 1.693‐fold; 95% CI: 1.092–2.624; *p* = 0.018) than those harbouring the WT gene (Table 4). The other two DPP4 SNPs, rs3788979 and rs6741949, showed no significant associations with clinicopathologic features mentioned above (data not shown).

**TABLE 3:** Odds ratio (OR) and 95% confidence interval (CI) of the clinical status and DPP4 rs7608798 genotypic frequencies in 704 patients with prostate cancer.

Variable	Genotypic frequency			
rs7608798	AA (*N* = 288)	AG + GG (*N* = 416)	OR (95% CI)	*p* value
PSA at diagnosis (ng/mL)				
≤10	131 (45.5%)	203 (48.8%)	1.00	0.387
>10	157 (54.5%)	213 (51.2%)	0.875 (0.648–1.183)	
Pathologic Gleason grade group				
1+2+3	240 (83.3%)	344 (82.7%)	1.00	0.824
4+5	48 (16.7%)	72 (17.3%)	1.047 (0.701–1.562)	
Clinical T stage				
1+2	258 (89.6%)	348 (83.7%)	1.00	**0.025** *
3+4	30 (10.4%)	68 (16.3%)	**1.680 (1.062–2.659)**	
Pathologic T stage				
2	150 (52.1%)	222 (53.4%)	1.00	0.738
3+4	138 (47.9%)	194 (46.6%)	0.950 (0.703–1.283)	
Pathologic N stage				
N0	266 (92.4%)	378 (90.9%)	1.00	0.485
N1	22 (7.6%)	38 (9.1%)	1.215 (0.703–2.103)	
Seminal vesicle invasion				
No	233 (80.9%)	320 (76.9%)	1.00	0.206
Yes	55 (19.1%)	96 (23.1%)	1.271 (0.876–1.844)	
Perineural invasion				
No	72 (25.0%)	114 (27.4%)	1.00	0.477
Yes	216 (75.0%)	302 (72.6%)	0.883 (0.627–1.244)	
Lymphovascular invasion				
No	241 (83.7%)	351 (84.4%)	1.00	0.804
Yes	47 (16.3%)	65 (15.6%)	0.950 (0.630–1.430)	
D'Amico classification				
Low risk/ Intermediate risk	151 (52.4%)	198 (47.6%)	1.00	0.207
High risk	137 (47.6%)	218 (52.4%)	1.214 (0.898–1.639)	

*Note*: The ORs with their 95% CIs were estimated by logistic regression models.

Abbreviations: N, node; PSA, prostate‐specific antigen; T, tumour.

* Bold values indicate *p* < 0.05 as statistically significant.

**TABLE 4:** Odds ratio (OR) and 95% confidence interval (CI) of the clinical status and DPP4 rs2268889 genotypic frequencies in 704 patients with prostate cancer.

Variable	Genotypic frequency			
rs2268889	TT (*N* = 389)	TC + CC (*N* = 360)	OR (95% CI)	*p*‐Value
PSA at diagnosis (ng/mL)				
≤10	155 (45.1%)	179 (49.7%)	1.00	0.215
>10	189 (54.9%)	181 (50.3%)	0.829 (0.617–1.115)	
Pathologic Gleason grade group				
1+2+3	282 (82.0%)	302 (83.9%)	1.00	0.500
4+5	62 (18.0%)	58 (16.1%)	0.874 (0.590–1.294)	
Clinical T stage				
1+2	307 (89.2%)	299 (83.1%)	1.00	0.018*
3+4	37 (10.8%)	61 (16.9%)	1.693 (1.092–2.624)	
Pathologic T stage				
2	182 (52.9%)	190 (52.8%)	1.00	0.973
3+4	162 (47.1%)	170 (47.2%)	1.005 (0.748–1.352)	
Pathologic N stage				
N0	314 (91.3%)	330 (91.7%)	1.00	0.854
N1	30 (8.7%)	30 (8.3%)	0.952 (0.561–1.615)	
Seminal vesicle invasion				
No	277 (80.5%)	276 (76.7%)	1.00	0.213
Yes	67 (19.5%)	84 (23.3%)	1.258 (0.876–1.807)	
Perineural invasion				
No	86 (25.0%)	100 (27.8%)	1.00	0.403
Yes	258 (75.0%)	260 (72.2%)	0.867 (0.619–1.212)	
Lymphovascular invasion				
No	285 (82.8%)	307 (85.3%)	1.00	0.378
Yes	59 (17.2%)	53 (14.7%)	0.834 (0.557–1.250)	
D'Amico classification				
Low risk/Intermediate risk	175 (50.9%)	174 (48.3%)	1.00	0.501
High risk	169 (49.1%)	186 (51.7%)	1.107 (0.824–1.488)	

*Note*: The ORs with their 95% CIs were estimated by logistic regression models.

Abbreviations: N, node; PSA, prostate‐specific antigen; T, tumour.

* Bold values indicate *p* < 0.05 as statistically significant.

### 3.4 Downregulation of DPP4 in PCa tissues correlates with tumour progression and a poor prognosis

**FIGURE 1:**
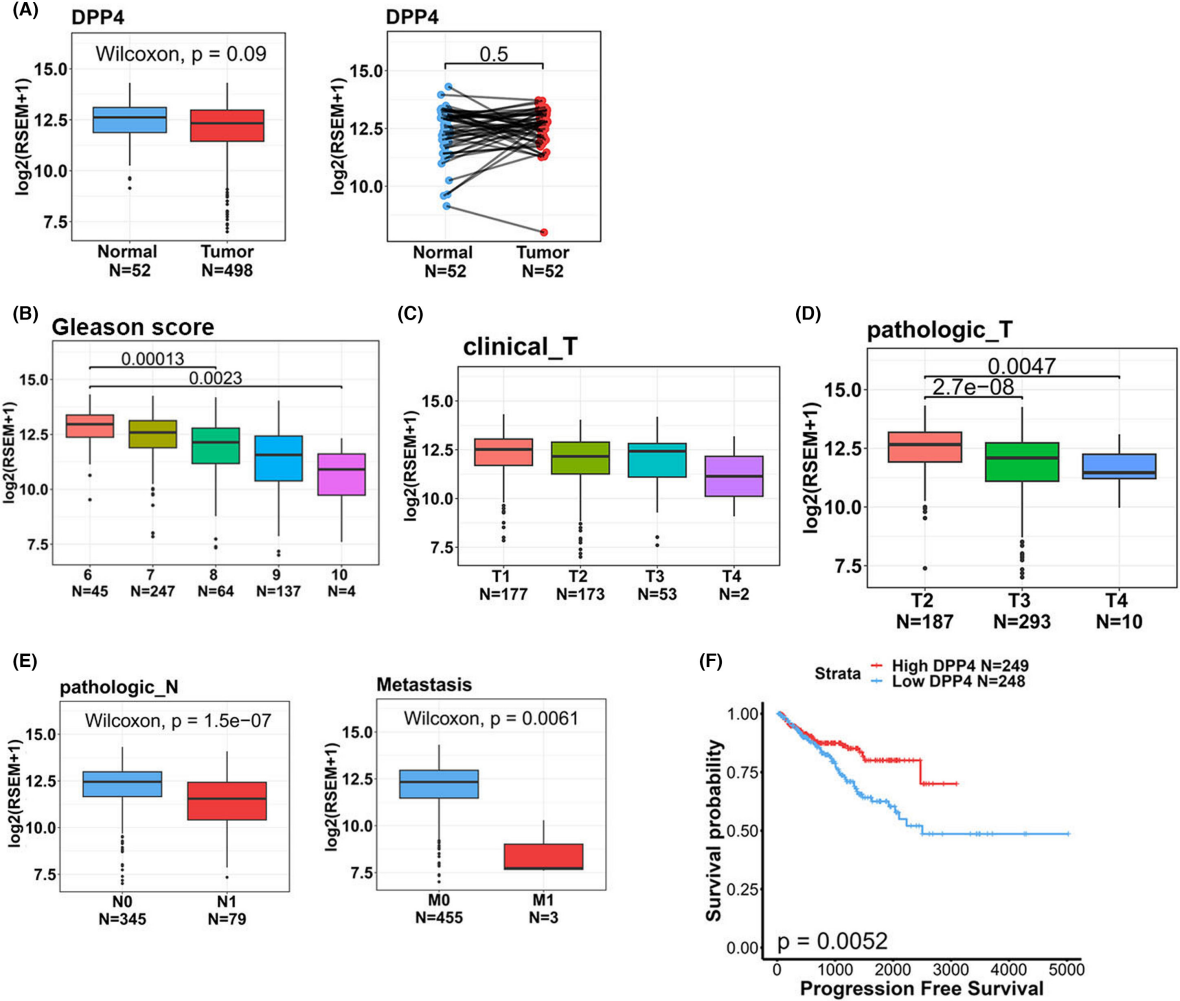
Clinical relevance of dipeptidyl peptidase IV (DPP4) levels in prostate cancer (PCa) patients obtained from TCGA database. (A) DPP4 gene expression in paired adjacent (right panel) and unpaired (left panel) normal and tumour tissues derived from patients with PCa (database source: TCGA‐prostate adenocarcinoma (PRAD)). (B–E) DPP4 gene expression levels in PCa from TCGA were compared according to the Gleason score (B), clinical T stage (C), pathological T stage (D), and lymph node and distal metastasis (E). (F) Kaplan–Meier curves for progression‐free survival of patients with PCa, as categorized according to high or low expression of DPP4. The *p* value indicates a comparison between patients with DPP4^high^ and DPP4^low^ (database source: TCGA‐PRAD).

To further analyse DPP4 expression levels in normal and PCa tissues and examine correlations of DPP4 levels with progression and prognosis of PCa, we utilized TCGA‐PRAD dataset and observed no significant difference in DPP4 expression levels between tumour and noncancerous tissues (Figure 1A, left panel) or tumour and corresponding matched normal tissues (Figure 1A, right panel). However, we observed that relative levels of DPP4 transcripts were lower in PCa patients with a high Gleason score (Figure 1B), advanced cT (Figure 1C), pathological T stages (Figure 1D) and lymph node (Figure 1E, left panel) or distal (Figure 1E, right panel) metastasis. Moreover, a Kaplan–Meier plot revealed that PCa patients from TCGA‐PRAD dataset with DPP4^low^ tumours had shorter PFS times compared to those with DPP4^high^ tumours (Figure 1F).

### 3.5 Overexpression of DPP4 suppresses migration and invasion of PCa cells

**FIGURE 2:**
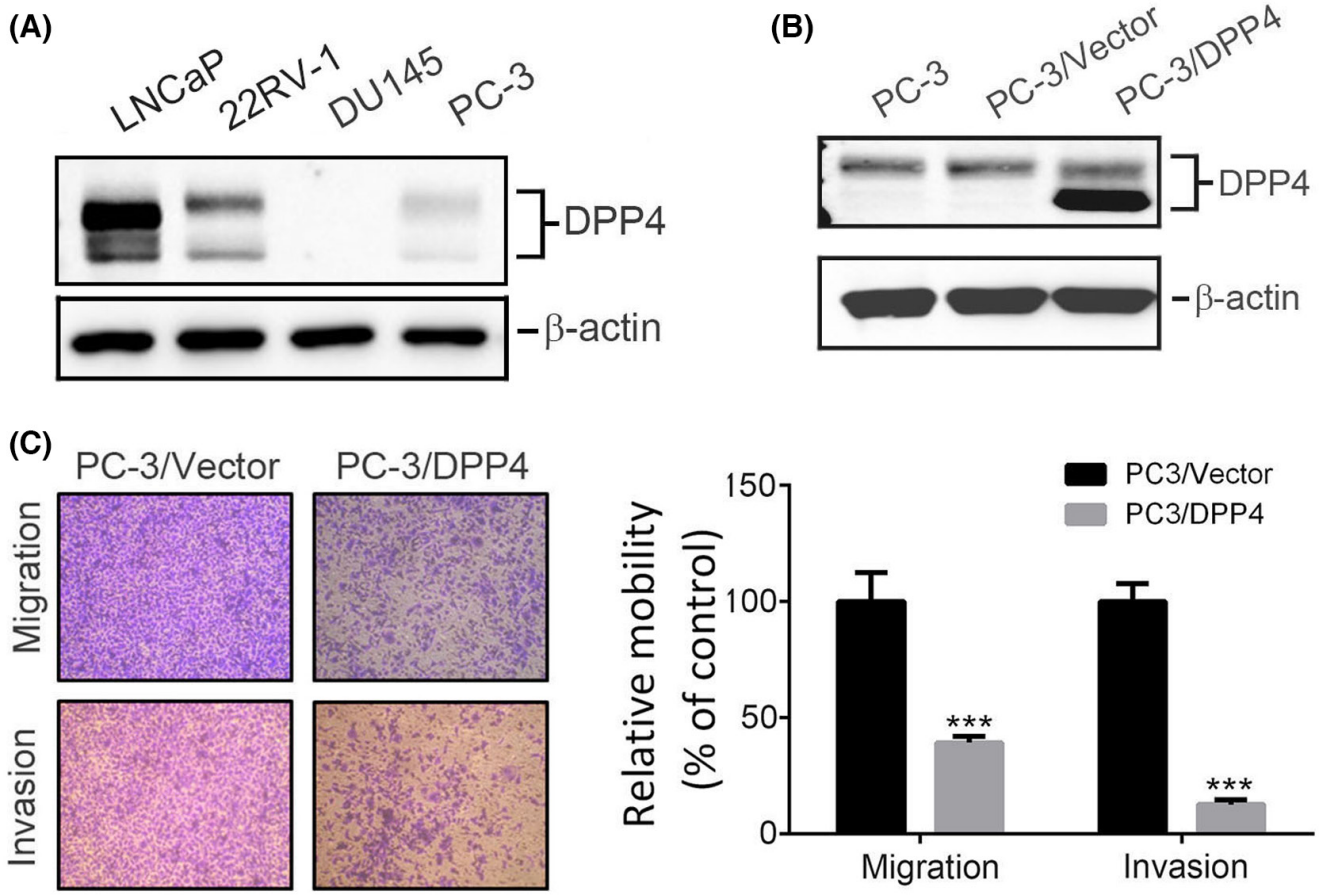
Overexpression of dipeptidyl peptidase IV (DPP4) suppresses migratory and invasive abilities of prostate cancer (PCa) cells. (A) Protein levels of DPP4 were determined by Western blotting in a set of PCa cell lines including LNCaP (androgen‐dependent), 22RV‐1 (non‐metastatic, androgen‐independent), and PC3 and DU145 (metastatic, androgen‐independent). (B and C) PC3 cells overexpressed DPP4 by transiently transfection with the pENTER‐DPP4 plasmid or a control vector (B) and subjected to migration and invasion assays (C). DPP4 overexpression suppressed both the migratory and invasive abilities of PC3 cells. Quantitative results by counting invaded cells in a 100× field. Multiples of differences are presented as the mean ± SD of three independent experiments. *** *p* < 0.001, compared to the vector control group.

In in vitro study, we first detected DPP4 expression levels in a set of PCa cell lines including LNCaP (androgen‐dependent), 22RV‐1 (non‐metastatic, androgen‐independent), and PC3 and DU145 (metastatic, androgen‐independent) cells using Western blotting. We observed that PC3 and DU145 cells expressed lower DPP4 protein levels compared to LNCaP or 22RV‐1 cells (Figure 2A). Next, to determine whether DPP4 modulates the cell migratory and invasive abilities, we overexpressed DPP4 in PC3 cells (Figure 2B). We found that regardless of the migratory or invasive abilities of PC3 cells, all were significantly attenuated by DPP4 overexpression compared to control cells (Figure 2C). Taken together, these clinical data and in vitro cell studies imply that DPP4 may play a tumour‐suppressive role in the progression of PCa, and DPP4 SNPs might affect PCa progression through influencing DPP4 expression.

## 4 DISCUSSION

Previous studies indicated the impact of DPP4 genetic variants on several diseases. For example, the DPP4 rs3788979 SNP may have a cardioprotective effect and prove to be a useful predictor for evaluating the severity of coronary stenosis in Chinese patients with cardiovascular disease and type 2 diabetes mellitus (T2DM).^20^ The rs3788979 SNP was also reported to be correlated with a high risk of coronavirus disease 2019 (COVID‐19) disease, and carriers of the TT genotype had the lowest DPP4 levels.^21^ Both of the rs3788979 and rs7608798 SNPs were significantly related to a risk of T2DM in an Indian population.^22^ Compared to T2DM, very few studies have addressed impacts of DPP4 genetic variants on cancer risk or progression.^23^ Higashibata et al. indicated that the DPP4 rs3788979 or rs7608798 SNP influenced baseline PSA levels in healthy Japanese men aged 60–69 years, implying that DPP4 SNPs might affect prostate carcinogenesis in elderly Japanese men.^27^ However, knowledge of the clinical relevance of DPP4 SNPs in PCa, which probably result in expression and functional changes of DPP4, is still lacking. Herein, we found for the first time that DPP4 genetic variants play critical roles in influencing the clinicopathological characteristics of PCa in a Taiwanese population.

Our present data indicated that patients with a mutant base G of rs7608798 or base C of rs2268889 had a significantly higher risk of developing an advanced cT stage (3+4) under a dominant model (AG+GG and TC+CC). cT stages of T3 and T4 indicate that the tumour has grown outside the prostate and may spread to seminal vesicles (T3) and other distant organs such as the rectum and bladder (T4). These results were similar to observations of our previous study, which indicated that oral cancer patients without a history of cigarette smoking and who had at least one mutant C allele of DPP4 rs2268889 had a significantly higher frequency of developing an advanced T stage.^23^ To the best of our knowledge, the roles of DPP4 in cancers are diverse, as its function in tumour development varies depending on the tumour type. DPP4 can be either up‐ or downregulated in various cancer types.^13^ For example, DPP4 transcripts from hepatocellular carcinoma (HCC) were significantly increased and were associated with a significantly larger tumour size. DPP4‐knockdown by small interfering (si)RNA in vitro led to suppression of tumour growth through cell cycle arrest in HCC cell lines, suggesting that DPP4 has a pro‐oncogenic role in HCC.^28^ In contrast, DPP4 was downregulated during the malignant transformation to melanoma cells, and DPP4 overexpression decreased the invasive ability of melanoma cells, implying an anti‐oncogenic role of DPP4 in melanoma.^29^ In PCa, we observed that DPP4 transcript levels were comparable in normal and PCa tissues, but inversely correlated with the Gleason score, cT stage, pathological T stage and metastasis. Moreover, DPP4 expression was correlated with a longer PFS in PCa patients. In PCa cell lines, we found that DPP4 was highly expressed in androgen‐sensitive LNCaP PCa cells but barely expressed in androgen‐insensitive and metastatic DU145 and PC3 PCa cells. DPP4 overexpression significantly suppressed the migratory and invasive abilities of PC3 cells. Our clinical and in vitro studies all indicated that DPP4 has an anti‐oncogenic role in PCa. In addition to PCa, the tumour‐suppressive role of DPP4 was also demonstrated in oral cancer, as evidenced by the fact of a reduction in migration of DPP4‐overexpressing SCC9 oral cancer cells (Figure S1). Taken together, we suggest that the DPP4 rs7608798 or rs2268889 SNP might result in decreased DPP4 expression and subsequently cause progression of PCa and oral cancer.

Both rs7608798 or rs2268889 SNPs are located in the intron regions of DPP4. Intronic SNPs influence genetic susceptibility to cancer through both genetic and epigenetic mechanisms. For instance, intronic sequences contain many cis‐acting regulatory elements, such as transcription factors, enhancers, and silencers that can positively or negatively regulate gene expression.^30^ For example, three SNPs, rs2981578, rs45631563 and rs35054928, of fibroblast growth factor receptor 2 (FGFR2) are mapped to transcriptional silencer elements and enhance silencer activity, resulting in lower FGFR2 expression and increased risk of breast cancer.^31^ The rs12343867 T>C SNP in intron 14 of Janus‐activated kinase 2 (JAK2) is associated with myeloproliferative neoplasms by acting as a transcriptional repressor.^32^ In addition, many non‐coding RNA motifs genes, such as long noncoding (lnc)RNA are located in intronic sequences, and intronic lncRNAs have been extensively identified to be functional in regulating expressions of their corresponding host genes.^33^ Whether the rs7608798 or rs2268889 SNPs are located in any intronic lncRNA region remains unknown. The transcriptional potential of SNP rs7608798 A>G and rs2268889 T>C in the DPP4 intron will be further investigated in our future work.

Nevertheless, this study still has some limitations that need to be discussed. First, acknowledging that the current study had a relatively small sample size is essential, as it may limit the statistical power and precision of the results. Conducting larger independent cohorts from different medical centres can provide more robust and reliable findings regarding the impact of DPP4 SNPs on the risk and development of PCa. Second, recognizing that the study was conducted in the Taiwanese population, it is crucial to explore whether the observed results can be generalized to other ethnic groups. Including other ethnic populations in future studies will allow for comparisons and validation of the findings across different races. Third, our study cannot determine the influences of DPP4 SNPs on DPP4 expression, so the mRNA and DNA should be collected simultaneously from the same samples to further validate this issue in future work. Last, acknowledging that the study focused solely on the association of DPP4 SNPs with PCa, the impacts of genetic variations in its closely related genes, such as DPP8 and DPP9, should be further investigated.

In conclusion, we identified diverse allelic effects of DPP4 SNPs (rs7608798 and rs2268889) in a Taiwanese population, which affected the clinicopathologic development of PCa. We also confirmed the tumour‐suppressive role and prognostic effect of DPP4 in PCa using PCa clinical samples and PCa cell lines. Our results suggested that the DPP4 rs7608798 and rs2268889 SNPs might influence DPP4 gene expression and subsequently promote PCa progression. The DPP4 rs7608798 and rs2268889 polymorphisms may be pivotal markers to predict PCa tumour aggressiveness and prognosis.

## AUTHOR CONTRIBUTIONS

**Yu‐Ching Wen:** Conceptualization (equal); data curation (equal); funding acquisition (equal); writing – original draft (equal). **Chia‐Yen Lin:** Data curation (equal); resources (equal). **Chi‐Hao Hsiao:** Methodology (equal). **Shian‐Shiang Wang:** Data curation (equal); resources (equal). **Hsiang‐Ching Huang:** Methodology (equal); software (equal). **Yung‐Wei Lin:** Conceptualization (equal). **Kuo‐Hao Ho:** Data curation (equal); software (equal). **Lun‐Ching Chang:** Software (equal). **Shun‐Fa Yang:** Conceptualization (equal); methodology (equal); writing – original draft (equal). **Ming‐Hsien Chien:** Conceptualization (equal); funding acquisition (equal); software (equal); writing – original draft (equal); writing – review and editing (equal).

## CONFLICT OF INTEREST STATEMENT

The authors declare no conflicts of interest related to this study.

## Supporting information


Data S1:

## Data Availability

The data used to support the findings of this study are available from the corresponding author upon request.

## References

[1] Siegel RL , Miller KD , Wagle NS , Jemal A . Cancer statistics, 2023. CA Cancer J Clin. 2023;73(1):17‐48. 36633525 10.3322/caac.21763

[2] Lin PH , Chang SW , Tsai LH , et al. Increasing incidence of prostate cancer in Taiwan: a study of related factors using a nationwide health and welfare database. Medicine (Baltimore). 2020;99(39):e22336. 32991446 10.1097/MD.0000000000022336 PMC7523769

[3] Bo M , Ventura M , Marinello R , Capello S , Casetta G , Fabris F . Relationship between prostatic specific antigen (PSA) and volume of the prostate in the benign prostatic hyperplasia in the elderly. Crit Rev Oncol Hematol. 2003;47(3):207‐211. 12962896 10.1016/s1040-8428(03)00094-5

[4] Wang G , Zhao D , Spring DJ , DePinho RA . Genetics and biology of prostate cancer. Genes Dev. 2018;32(17–18):1105‐1140. 30181359 10.1101/gad.315739.118 PMC6120714

[5] Shastry BS . SNP alleles in human disease and evolution. J Hum Genet. 2002;47(11):561‐566. 12436191 10.1007/s100380200086

[6] Chan EY . Next‐generation sequencing methods: impact of sequencing accuracy on SNP discovery. Methods Mol Biol. 2009;578:95‐111. 19768588 10.1007/978-1-60327-411-1_5

[7] Dell'atti L , Aguiari G . The role of genetic polymorphisms in the diagnosis and Management of Prostate Cancer: an update. Anticancer Res. 2023;43(1):317‐322. 36585200 10.21873/anticanres.16166

[8] Benafif S , Kote‐Jarai Z , Eeles RA . A review of prostate cancer genome‐wide association studies (GWAS). Cancer Epidemiol Biomarkers Prev. 2018;27(8):845‐857. 29348298 10.1158/1055-9965.EPI-16-1046 PMC6051932

[9] Vieira GM , Gellen LPA , da Veiga Borges Leal DF , et al. Correlation between genomic variants and worldwide epidemiology of prostate cancer. Genes (Basel). 2022;13(6):1039. 35741800 10.3390/genes13061039 PMC9222668

[10] de Souza MR , de Souza MF , de Nóbrega M , et al. Polymorphic variants of the CASP3, CASP9, BCL‐2 and NKX3‐1 genes as candidate markers for prostate cancer susceptibility and poor prognosis. Mol Biol Rep. 2022;49(9):9079‐9087. 35708863 10.1007/s11033-022-07654-0

[11] Shiota M , Fujimoto N , Imada K , et al. Potential role for YB‐1 in castration‐resistant prostate cancer and resistance to enzalutamide through the androgen receptor V7. J Natl Cancer Inst. 2016;108(7):djw005. 10.1093/jnci/djw005 26857528

[12] Shiota M , Narita S , Habuchi T , Eto M . Validated prognostic significance of YB‐1 genetic variation in metastatic prostate cancer. Pharmacogenomics J. 2021;21(1):102‐105. 32963329 10.1038/s41397-020-00188-3

[13] Enz N , Vliegen G , De Meester I , et al. CD26/DPP4–a potential biomarker and target for cancer therapy. Pharmacol Ther. 2019;198:135‐159. 30822465 10.1016/j.pharmthera.2019.02.015

[14] Deacon CF . Physiology and pharmacology of DPP‐4 in glucose homeostasis and the treatment of type 2 diabetes. Front Endocrinol (Lausanne). 2019;10:80. 30828317 10.3389/fendo.2019.00080 PMC6384237

[15] Wilson MJ , Ruhland AR , Quast BJ , Reddy PK , Ewing SL , Sinha AA . Dipeptidylpeptidase IV activities are elevated in prostate cancers and adjacent benign hyperplastic glands. J Androl. 2000;21(2):220‐226. 10714816

[16] Lu Z , Qi L , Bo XJ , Liu GD , Wang JM , Li G . Expression of CD26 and CXCR4 in prostate carcinoma and its relationship with clinical parameters. J Res Med Sci. 2013;18(8):647‐652. 24379839 PMC3872602

[17] Russo JW , Gao C , Bhasin SS , et al. Downregulation of dipeptidyl peptidase 4 accelerates progression to castration‐resistant prostate cancer. Cancer Res. 2018;78(22):6354‐6362. 30242112 10.1158/0008-5472.CAN-18-0687 PMC6239953

[18] Wesley UV , McGroarty M , Homoyouni A . Dipeptidyl peptidase inhibits malignant phenotype of prostate cancer cells by blocking basic fibroblast growth factor signaling pathway. Cancer Res. 2005;65(4):1325‐1334. 15735018 10.1158/0008-5472.CAN-04-1852

[19] Nazarian A , Lawlor K , Yi SS , et al. Inhibition of circulating dipeptidyl peptidase 4 activity in patients with metastatic prostate cancer. Mol Cell Proteomics. 2014;13(11):3082‐3096. 25056937 10.1074/mcp.M114.038836 PMC4223493

[20] Wang Z , Liu Y , Wang W , Qu H , Han Y , Hou Y . Association of dipeptidyl peptidase IV polymorphism, serum lipid profile, and coronary artery stenosis in patients with coronary artery disease and type 2 diabetes. Medicine (Baltimore). 2021;100(13):e25209. 33787603 10.1097/MD.0000000000025209 PMC8021284

[21] Posadas‐Sánchez R , Sánchez‐Muñoz F , Guzmán‐Martín CA , et al. Dipeptidylpeptidase‐4 levels and DPP4 gene polymorphisms in patients with COVID‐19. Association with disease and with severity. Life Sci. 2021;276:119410. 33774023 10.1016/j.lfs.2021.119410 PMC7989663

[22] Bhargave A , Devi K , Ahmad I , Yadav A , Gupta R . Genetic variation in DPP‐IV gene linked to predisposition of T2DM: a case control study. J Diabetes Metab Disord. 2022;21(2):1709‐1716. 36249912 10.1007/s40200-022-01131-y PMC9554862

[23] Chen PJ , Lu HJ , Nassef Y , et al. Association of dipeptidyl peptidase IV polymorphism with clinicopathological characters of oral cancer. J Oral Pathol Med. 2022;51(8):730‐737. 35880802 10.1111/jop.13337

[24] Lin YW , Wang SS , Wen YC , et al. Genetic variations of melatonin receptor type 1A are associated with the clinicopathologic development of urothelial cell carcinoma. Int J Med Sci. 2017;14(11):1130‐1135. 29104467 10.7150/ijms.20629 PMC5666544

[25] Lee WJ , Hsiao M , Chang JL , et al. Quercetin induces mitochondrial‐derived apoptosis via reactive oxygen species‐mediated ERK activation in HL‐60 leukemia cells and xenograft. Arch Toxicol. 2015;89(7):1103‐1117. 25138434 10.1007/s00204-014-1300-0

[26] Yang SF , Lee WJ , Tan P , et al. Upregulation of miR‐328 and inhibition of CREB‐DNA‐binding activity are critical for resveratrol‐mediated suppression of matrix metalloproteinase‐2 and subsequent metastatic ability in human osteosarcomas. Oncotarget. 2015;6(5):2736‐2753. 25605016 10.18632/oncotarget.3088 PMC4413614

[27] Higashibata T , Naito M , Mori A , et al. DPP4 genetic variants influence baseline prostate‐specific antigen levels: the J‐MICC study. Nagoya J Med Sci. 2013;75(1–2):73‐80. 23544270 PMC4345699

[28] Kawaguchi T , Kodama T , Hikita H , et al. Synthetic lethal interaction of combined CD26 and Bcl‐xL inhibition is a powerful anticancer therapy against hepatocellular carcinoma. Hepatol Res. 2015;45(9):1023‐1033. 25297967 10.1111/hepr.12434

[29] Pethiyagoda CL , Welch DR , Fleming TP . Dipeptidyl peptidase IV (DPPIV) inhibits cellular invasion of melanoma cells. Clin Exp Metastasis. 2000;18(5):391‐400. 11467771 10.1023/a:1010930918055

[30] Deng N , Zhou H , Fan H , Yuan Y . Single nucleotide polymorphisms and cancer susceptibility. Oncotarget. 2017;8(66):110635‐110649. 29299175 10.18632/oncotarget.22372 PMC5746410

[31] Campbell TM , Castro MAA , de Santiago I , et al. FGFR2 risk SNPs confer breast cancer risk by augmenting oestrogen responsiveness. Carcinogenesis. 2016;37(8):741‐750. 27236187 10.1093/carcin/bgw065 PMC4967216

[32] Spasovski V , Tosic N , Nikcevic G , et al. The influence of novel transcriptional regulatory element in intron 14 on the expression of Janus kinase 2 gene in myeloproliferative neoplasms. J Appl Genet. 2013;54(1):21‐26. 23188718 10.1007/s13353-012-0125-x

[33] Wu H , Yang L , Chen LL . The diversity of long noncoding RNAs and their generation. Trends Genet. 2017;33(8):540‐552. 28629949 10.1016/j.tig.2017.05.004

