# Supplementary material for: Genetic variants of dipeptidyl peptidase IV are linked to the clinicopathologic development of prostate cancer
Source: J Cell Mol Med. 2023 Aug 2;27(17):2507–16. doi: 10.1111/jcmm.17845 (PMC10468658; doi:10.1111/jcmm.17845)
Supplement: Supplementary file 1 — Data S1: [file JCMM-27-2507-s001.pdf]

Supplemental Information

Title: Genetic variants of dipeptidyl peptidase IV are linked to the clinicopathologic development of prostate cancer

Yu-Ching Wen, Chia-Yen Lin, Chi-Hao Hsiao, Shian-Shiang Wang, Hsiang-Ching Huang, Yung-Wei Lin, Kuo-Hao Ho, Lun-Ching Chang, Shun-Fa Yang*, and Ming-Hsien Chien*

*Correspondence to: Dr. Shun-Fa Yang (E-mail: ysf@csmu.edu.tw) and Dr. Ming-Hsien Chien (E-mail: mhchien1976@gmail.com)

Supplemental methods

Cell culture

The human SCC9 oral squamous cell carcinoma (OSCC) cell line was purchased from American Type Culture Collection (ATCC, Manassas, VA, USA). Cells were cultured in Dulbecco's modified Eagle medium/nutrient mixture F-12 (DMEM/F12; Life Technologies, Grand Island, NY, USA) containing 10% fetal bovine serum and maintained in an incubator at 37 °C with a 5% CO_2 and 95% air atmosphere.

Boyden chamber migration assay

The migration assay was conducted following methods described by Yang et al[1]. Briefly, SCC9/vector or SCC9/DPP4 cells were harvested and seeded into a Boyden chamber (Neuro Probe, Cabin John, MD, USA) at a density of 10^4 cells/well in serum-free medium. The bottom chamber was filled with standard medium that served as a chemoattractant. After 24 h of incubation, migratory cells were fixed with methanol, stained with 10% Giemsa (Sigma Chemical, St. Louis, MO, USA), and counted under a light microscope at 100× magnification, with three random fields per well.

Figure Legends

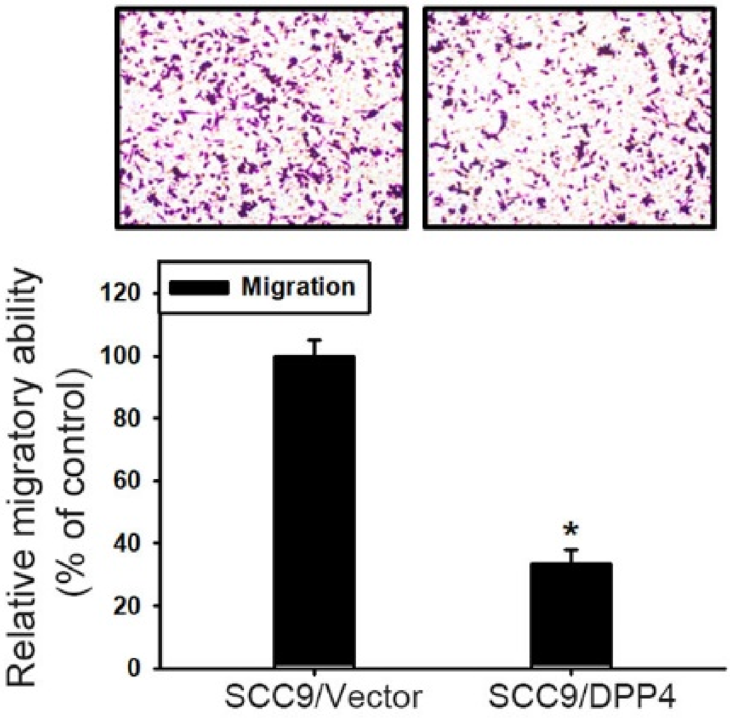

Figure S1. Effect of DPP4 overexpression on migration of SCC9 oral squamous cell carcinoma (OSCC) cells. SCC9 cells overexpressed DPP4 by transiently transfection with the pENTER-DPP4 plasmid or a control vector and were subjected to a Boyden chamber migration assay. Values are presented as the mean ± SD of three independent experiments. * $p < 0.05$ compared to the control group.

Reference

1 Yang SF, Chen MK, Hsieh YS et al. Antimetastatic effects of Terminalia catappa L. on oral cancer via a down-regulation of metastasis-associated proteases. *Food Chem Toxicol.* 2010; 48(4):1052-1058.
